# Use of Barcode Technology Can Make a DIfference to Patient Safety in the Post COVID era

**DOI:** 10.1093/ijcoms/lyab014

**Published:** 2021-08-03

**Authors:** Peter Lachman, Els van der Wilden-van Lier

**Affiliations:** Education Quality Improvement, Royal College of Physicians of Ireland, Frederick House, 19 South Frederick Street, Dublin 2, Ireland; GS1, Healthcare, Steenhoffstraat 30-26, Soest, Netherlands

**Keywords:** Patient Safety, COVID-19, Barcodes, Human Factors Supply Chain, Standards

## Abstract

The COVID- 19 pandemic has demonstrated the value of digital solutions to patient care and to patient safety. here are many solutions that have yet to be fully implemented. In this commentary we discuss the value of barcode technology to ensure secure supply chains and the delivery of reliable and efficient processes in healthcare. This will facilitate the implementation of WHO policies on supply chains as well as support initiatives on medication safety.

As we emerge from the COVID pandemic, we can reflect on the systems that we have in place to ensure the safety of people providing care and those receiving care. In this paper we propose that developing safe clinical systems will require the translation of safety theory into clinical practice, with the adoption of available technological solutions. Vincent and Amalberti have argued that we need to proactively manage the inherent risk that exists in healthcare.[1] Proactive management requires an assessment of how the work system actually operates and where improvements can be made. A Human Factors and Ergonomics (HFE) approach can assist in understanding the sociotechnical processes needed to ensure safe delivery of care and safe supply systems for care delivery.[2] At the same time, systems must be resilient to allow for constantly learning from its operations.[3] Unfortunately, it would appear that the healthcare system is often slow to adopt new technologies and interventions that are known to work in other industries. One could be tempted to look for new solutions. We believe that if we first reliably apply what we know works to improve safety, then we will be safer. The challenge is in the implementation of proven solutions.

The COVID-19 pandemic demonstrated that systems were not prepared, had variable resilience and did not proactively manage risk [4]. Perhaps the major problem is that the science of patient safety has not been uniformly applied across the health care systems and the available technology has not been implemented, despite clear benefit being demonstrated. Although we have many methods and tools based on scientific rigour, the application of the solutions has been problematic.[5]

Technologies such as the electronic medical record (EMR), computer physician order entry (CPOE), and barcode technology have been shown to have benefit when they are integrated into redesigned work systems, rather than as add-on solutions to existing designs. There have been calls for the adoption of the EMR, given its demonstrable benefits.[6] We believe that it should be accompanied by the adoption of barcode technology. This will ensure the integrity of supply chain systems, medical device management and clinical processes, such as medication management and work in the operating rooms and intensive care, where errors can easily be made. This will require a change in the way that we currently undertake many of our processes.

**Medication safety**

Medication safety is the current global patient safety challenge of the World Health Organisation. It is a complex set of sociotechnical interactions. To ensure a safer process it is possible to introduce technologies such as CPOE systems to improve prescribing, and robots to improve dispensing supported by technologies such as barcode scanning in procurement, dispensing, administration and reconciliation.

The economic impact of errors in the medication process is high.[7] A recent paper demonstrated the benefit of the latest barcode methodology to decrease medication harm.[8] This finding is not new and the safety benefits of this technology have been previously reported.[9,10] There has been progress mainly in dispensing[11], but the use of barcoding in the whole process of medication management has not been uniformly adopted. We believe that the adoption of barcode scanning, and the accessibility of it at scale with worldwide standards, can make a difference at relatively low cost.

Increasingly, regulation will drive the manufacturers to barcode their products, so many medical products will have barcode identification. This include GPS tracking to monitor the shipping of products, as well as barcodes to identify the product. For example, as the COVID-19 vaccines are new products being used widely, the GPS allows identification of where the product is in the distribution process and the barcode provides the identification and traceability of the individual vials or even doses. This is required to ensure safety, possible recalls and monitor surveillance, which has been important  when there has been an occurrence of side effects with several vaccines.

**Clinical area safety and supply control**

In clinical settings with complex processes and where invasive procedures are undertaken, the use of barcodes enhances the safety of these processes , from drug administration to the use of medical devices and instruments to the counting of surgical sponges. In high risk areas, such as the emergency room and intensive care, adding barcode technology can improve accountability, control of the supply chain and set standards of care at a higher level of reliability.

**Traceability and the supply chain**

The World Health Organisation recently published a policy paper on the importance of traceability, be it of medications or medical devices.[12] It is estimated that 10% of all medications in the LMIC are counterfeit. The introduction of the COVID-19 vaccine is a good example of how we must apply technology to ensure reliability in the process, from production to the distribution and then administration of the vaccine. The production of the vaccines at scale and then worldwide distribution requires accountability in origin and route of the supply. Barcoding technology can provide the public with reassurance of the safety of the vaccines and can ensure that there is reliability in the process. While the first COVID-vaccine-batches were not at all traceable, the vaccines are now traceable in Europe, in compliance with the EU Falsified Medicines Directive. As the spread of vaccines happens worldwide, the identification with barcodes will decrease the potential of counterfeit vaccines being used. Product identification with a globally unique identifier, with each batch having a unique identifier, allows for identification and traceability, should side-effects be an issue.

**Barriers and facilitators**

Despite the evidence that globally unique identification using barcode scanning can increase the reliability of medication safety and other clinical processes - and that this improves the efficacy of the supply chain, there appears to have been limited uptake within the health sector. There are few papers on the impact of the technology and some have indicated the challenges faced on implementation of barcode scanning.[13,14,15] Research is required, not into its efficacy but into how best barcode technology can be implemented at scale. A potential perceived problem would be justifying a Return on Investment (ROI) for implementation. Barcode technology is not restricted to hospital size, nor only to wealthier organisations in the private sector. The ROI can be demonstrated first on a smaller scale in a hospital, e.g. commencing in the operating room or pharmacy. The aim may be to decrease waste in the first instance, which would provide the ROI. Examples of a positive ROI are available in the Scan4Safety programme in the United Kingdom.[16]

If the WHO policy on traceability is to be successful, then healthcare will need to change how its processes are managed. This is very important when supply chains are disrupted due to natural challenges like the pandemic, as well as other challenges such as war and natural disasters, in which the reliable restoration of supplies is essential. Barcoding facilitates clear visibility of stock and clarity in planning.

We now have international standards for barcodes[17] and for healthcare organisations.[18] Barcode technology is not the sole answer to the challenge of supply control and patient safety. However, it is a tool that, within a culture of safety, can help to increase clinical and patient trust in the system processes. Together with proactive management of risk, HFE and the building of resilience, it can help to build a safer and more efficient healthcare system.

As we move to universal health coverage and the WHO Decade of Patient Safety, it is time to adopt a technology that has existed for many decades and that can make care safer and more efficient.

## Supplementary Material

lyab014_Supp

